# CourseQ: the impact of visual and interactive course recommendation in university environments

**DOI:** 10.1186/s41039-021-00167-7

**Published:** 2021-06-30

**Authors:** Boxuan Ma, Min Lu, Yuta Taniguchi, Shin’ichi Konomi

**Affiliations:** 1 Graduate School of Information Science and Electrical Engineering, Kyushu University, Fukuoka, Japan; 2 Faculty of Arts and Science, Kyushu University, Fukuoka, Japan; 3 Research Institute for Information Technology, Kyushu University, Fukuoka, Japan

**Keywords:** Course recommendation, University, Interactive system, Visualization

## Abstract

The abundance of courses available in a university often overwhelms students as they must select courses that are relevant to their academic interests and satisfy their requirements. A large number of existing studies in course recommendation systems focus on the accuracy of prediction to show students the most relevant courses with little consideration on interactivity and user perception. However, recent work has highlighted the importance of user-perceived aspects of recommendation systems, such as transparency, controllability, and user satisfaction. This paper introduces CourseQ, an interactive course recommendation system that allows students to explore courses by using a novel visual interface so as to improve transparency and user satisfaction of course recommendations. We describe the design concepts, interactions, and algorithm of the proposed system. A within-subject user study (N=32) was conducted to evaluate our system compared to a baseline interface without the proposed interactive visualization. The evaluation results show that our system improves many user-centric metrics including user acceptance and understanding of the recommendation results. Furthermore, our analysis of user interaction behaviors in the system indicates that CourseQ could help different users with their course-seeking tasks. Our results and discussions highlight the impact of visual and interactive features in course recommendation systems and inform the design of future recommendation systems for higher education.

## Introduction

Recommendation systems are increasingly used in many everyday services; they exploit very large information spaces to personalize many aspects of our digital lives and to help alleviate the problem of information overload in a variety of domains. In the education domain, course recommendation systems can make learning environments more adaptive and effective by alleviating certain types of information overlord (Ma et al. 2020). The course recommendation in universities, however, is different from the conventional movie recommendation or music recommendation because of the unique characteristics of educational settings such as course enrollment.

First, compared with the conventional recommendation systems, course recommendation systems in university environments can suffer from the cold start problem more severely. Every year, freshmen need to navigate their new academic environments, but the historical course-enrollment information of freshmen is often too limited. Without sufficient information, traditional recommendation algorithms such as collaborative filtering would only yield very coarse recommendation results (Jing and Tang 2017). Second, traditional recommendation systems focus on recommending items that align with users’ interests, but course choice behaviors are not based purely on the interests of students. For example, some students would not enroll in courses whose content is of their interest, they may rather choose the courses that allow them to earn credits easily. Besides, students’ interests and goals can change as they explore and learn new things; their preferences extracted from historical data may differ from their current interests. In addition, other factors also play a part in the course selection process, such as social factors (Potts et al. 2018; Tinto 1997; Osborne et al. 2003). Third, watching movies typically requires 2 h, and listening to music may require just a few minutes. In contrast, university courses usually last for several weeks and each class demands a lot of students’ attention. Thus, the cost for students to make a wrong decision is much higher for course recommendations than movie or music recommendations and it can have a long-lasting impact on students as improperly selecting courses would seriously affect their course experience, performance, and achievements and even cause students to drop out (Huang et al. 2019). Finally, students may have different information needs for using a course recommendation system and one recommendation strategy may not suit all students (Jiang et al. 2019). For example, some students may have general interests without a clear idea of what they want to study. For those students, course recommendations that help to explore various candidate courses can be extremely important. In contrast, the students who have clear learning goals would prefer narrowed-down results according to their goals and interests. They would appreciate specific and accurate results.

Given the importance of appropriate course selection for students, we need to consider the above challenges when building a useful course recommendation system for university environments. A key issue in addressing those problems is the provision of effective communication environments between users and computational intelligence by designing for proper interactivity and intelligibility. By involving students in the recommendation processes, the system could capture the user’s preferences interactively. The importance of interactive recommendation systems has been highlighted by researchers recently. They started to focus on user aspects, including transparency, trust, control, user’s interaction behavior, and general user experiences in recommendation systems (Pu et al. 2012; Alkan et al. 2019). It has been shown that users are interested not only in receiving precise recommendations but also in having a more active role in the entire recommendation process (Xiao and Benbasat 2007), and users may be willing to invest more effort to explore and even accept less accurate recommendations if they are able to have more influence over the system (Konstan and Riedl 2012). Compared to other domains, involving students in the recommendation processes becomes more important in the educational domain. However, there is a scarcity of previous work that integrates interaction into course recommendation systems. As a result, we consider course recommendation as an opportunity to truly engage the user in an interactive recommendation system.

In this context, we present an interactive course recommendation system by combining topic model-based visualization techniques with different recommendation techniques to support effective interaction, explanations, and control through such visualization. Our approach can increase user engagement with the system and allow users to flexibly explore large-item spaces while providing a high level of user control and transparency. Also, we believe that our approach can contribute to the realization of more effective course recommendation systems in university environments compared to previous works that only focus on the accuracy of recommendations. This paper is an extension of the work originally presented in Ma et al. (2021). Compared to our previous work, here we present a detailed description of the design choices made in the development of our interface and more comprehensive analysis of results to investigate the impact of visualization and interaction on the course recommendation system, and the key factors that influence the success of such a system.

The remainder of this article is organized as follows. First, we will review the related work. Then, we describe the CourseQ interface, visualizations, and interactions, followed by the descriptions of relevant technical details. Next, we introduce the user study and discuss the key results. Finally, we discuss the implication of our experimental results for the design of future interactive course recommendation systems.

## Related works

### Interactive recommendation

Based on the user’s explicit or implicit preferences, current recommendation systems often produce recommendations that fit the user’s requirements automatically, trying to reduce the user’s interaction effort and cognitive load (He et al. 2016; Ricci et al. 2011). On the one hand, since such recommendation systems require the availability of user-specific preference information, they suffer from the cold start problem. That is, they cannot make effective recommendations for new users or for new items that have no available information (Burke 2010). On the other hand, such recommendation systems usually afford little user interaction, which is difficult for users to give feedback thus may exacerbate the filter bubble effects (Tintarev and Masthoff 2011; Pariser 2011). Besides, those systems often work as a “black box”, i.e., recommendations are presented to the users, but they do not offer the user any insight into the system logic, and the rationale is not explained to end-users (Sinha and Swearingen 2002). The lack of transparency may hinder users in comprehending why an item is recommended and can lead to trust issues when recommendations fail (Herlocker et al. 2000). The effectiveness of recommendation systems cannot be considered merely based on recommendation accuracy (Swearingen and Sinha 2001). Thus, the potential of interactive recommendation approaches has been emphasized.

Many interactive recommendation systems are developed by combining visualization techniques to support transparency and controllability of the recommendation processes Du et al. (2017, 2019). Visualizing the recommendation results can strongly influence users’ understanding of complex data and help reduce cognitive efforts (Zudilova-Seinstra et al. 2009; Chi 2004; Parra et al. 2014). PeerChooser (O’Donovan et al. 2008) is a collaborative filtering recommendation engine with an interactive visualization interface. They use a node-link diagram and distance between nodes to indicate the similarity between different items. The visualization explains the recommendation algorithm and users could control the results by interacting with the system. SmallWorlds (Gretarsson et al. 2010) visualizes the inner logic of collaborative filtering recommendations in Facebook that allows users to specify, refine, and build item-preference profiles to generate useful items. Their user study shows that the visual interactive approach can help recommendation systems to generate better results with a good user experience. We believe such an approach is also useful in the education field. Due to the distinctive characteristic of course selection, having a good understanding of why they should take the course is important to help students with the decision process. Therefore, we apply visualization in course recommendation to help students knowing the reason why the course is recommended, which increase student’s trust in the system, improve their understanding of the course content and knowledge structure, and persuade them to accept the course. Besides, such visualization would engage students to interact more with the system.

Existing interactive recommendation systems have also been designed by allowing user intervention into the recommendation processes. Their applications allow users to play an active role by iteratively controlling the recommendation processes and refining the result set towards their requirements. For example, users could give feedback on the recommendation results by rating, removing, or sorting recommended items. Some systems allow users to edit their profile data or other input data sources which will be used by the recommendation algorithm. Also, users could control the recommendation process by choosing different criteria and changing the influence of selected criteria. Research shows that users tend to be more satisfied when they have control over how recommendation systems make suggestions for them. TasteWeights (Bostandjiev et al. 2012) is an interactive music recommendation system that allows users to control the impact of different algorithms as well as various input data sources to generate recommendation results. By weighting the influence of different information types and different data sources, users could have a better understanding of how the results were produced and recommendation accuracy has been improved. LinkedVis (Bostandjiev et al. 2013) uses the same visualization approach as TasteWeights, but in a different context and with different data sources. SetFusion (Parra and Brusilovsky 2015) visualizes relationships among recommended items and multiple recommendation techniques with a Venn diagram and color cues. These color cues are used to represent the different techniques of their hybrid recommendation system and are used to link the recommendation results to the techniques that produced these recommendations. Users could control the process of fusing or integrating different algorithms individually. Their research indicates that such an interactive recommendation with high user controllability resulted in increased user engagement and a better user experience. Jin et al. (2017) designed an interactive music recommendation system to investigate the effects of user control on recommendations. Their results show that the recommendations are more likely to be accepted by users if the system offers a higher level of user control.

Other interactive recommendation systems support user exploration that enables users to navigate through the information space to find other relevant items. MoodPlay (Andjelkovic et al. 2016; 2019) is a music recommendation system that integrates content and mood-based filtering in an interactive interface. The system allows the exploration of a music collection through latent emotional dimensions, thereby improving acceptance and understanding of recommendations. Labor Market Explorer (Gutiérrez et al. 2019) is another example of the interactive recommendation system, which enables job seekers to explore the labor market in a personalized way based on their skills and competencies. Those works propose a good solution for dealing with cold start problem and provide guidance in educational course selections.

It has also been shown that interactive recommendation systems have the potential to increase the diversity of content (Loepp et al. 2015). Tsai and Brusilovsky (2018) designed a recommendation interface to help users explore the different relevance prospects of recommended items and to stress their diversity. The Diversity Donut (Wong et al. 2011) is an interactive recommendation system that allows a user to control the level of opinion diversity and the coverage of recommendations.

As suggested by previous research on interactive recommendation systems, interactive visualizations can reduce user’s cognitive efforts, increase user engagement and acceptance, and improve user experience. However, those works are limited to traditional recommendations such as movies or music, which may significantly differ from the course recommendation in education field. The impact of visual and interaction of course recommendation has received little attention. Therefore, we propose our approach and believe the possibility of it to support better exploration, understanding, and user control in educational recommendation systems.

### Course recommendation

Recommending courses to students is a fundamental and challenging task in the university environment. Students are typically required to select the courses they will take from the many courses provided for the coming semester. Various approaches have been used for course recommendations in this context. By learning from historical enrollment data, they focus on recommending courses to students and thereby helping them complete their degrees successfully.

Some approaches focus on recommending courses by matching their interests and course information. Morsomme and Alferez (2019) used a topic model to provide a more flexible and realistic interpretation of a student’s interests and how they change over time. Then, a content-based match algorithm is used to recommend courses whose content best matches the student’s academic interests. Jing and Tang (2017) also used a topic model to train user latent information from their historical access behaviors to course pages to represent their learning interest. Then, their interest preference could be used to calculate the similarity, and a collaborative filtering approach is used to provide recommendations. Although use topic models, these systems often behave like a “black box" and do not give explanations and details of topics and terms that would allow students to reflect on their course selection. In contrast, we design our system using topic visualization (Kucher et al. 2018; Chatzimparmpas et al. 2020) to help users to understand topics, corresponding terms, and the relationships of different courses content.

Several course recommendation systems have been developed by mining relationships and discovering sequences from historical data. Aher and Lobo (2013) used association rule mining based on frequent patterns to extract interesting relations between courses from the data that describe students’ previous course selections. Together with the clustering method, the system recommends courses based on rules that they extracted from historical course enrollment data. Bendakir and Aïmeur (2006) also presented a course recommendation system based on association rules. They use user ratings in their recommendation system together with association rules to improve the result. Polyzou et al. (2019) proposed Scholars Walk, which uses a random-walk approach and captures the sequential relationships between different courses to provide recommendations. Recently, representation learning has been used in this domain. Pardos and Jiang (2020) introduced a novel modification to the skip-gram model and applied it to historic course enrollment sequences to learn course vector representations. The course vectors are then used to diversify recommendations based on the similarity to a student’s favorite course. Pardos et al. (2019) also proposed a course2vec model that uses neural network architecture. It takes multiple courses as input and a probability distribution over the courses as outputs, which is used for the recommendation. However, those systems often rely on historical enrollment data to train their model. Therefore, the performance can be significantly affected if the available data is not enough. Besides, those systems do not support exploration, which is particularly important in the context where students go through a broad exploratory phase before specializing. Compared to those systems, our approach could support exploration through visualization and better solve the cold-start problem.

Other works have focused on models of predicting outcomes in future courses based on what courses students took already and the grades they earned (Elbadrawy et al. 2015). Those systems recommend the courses that students can pass easily or get relatively high grades (Hu and Rangwala 2018). Sweeney et al. (2016) developed a system using a hybrid of the Random Forest model and the MF-based Factorization Machine for next-term student grade prediction. Elbadrawy and Karypis (2016) addressed the grade prediction and top-n course ranking problems by using student and course academic features. Those methods help students be successful and graduate in a timely manner, but they also suffer from the cold-start problem when recommending courses for a freshman who has no available data. Besides, it can be prone to only recommending easier courses without considering the content of courses and student interest (Morsy and Karypis 2019).

Despite the significant success of various course recommendations, the impact of visualization and interaction for course recommendations has received little attention. In contrast to the aforementioned approaches, we propose an approach that combines the course recommendation and visualization to build an effective interactive course recommendation interface. We applied topic modeling in our approach since it is adoptable to visualize and provides us a way to represent course content in more detail, which could improve student’s understanding of the course content and knowledge structure. The recommendation interface we proposed allows students to interactively improve the recommendations, which helps system avoid cold start problem and bring students own preferences to the system. Also, it has the benefit of allowing for the control of the recommendation processes, as well as the increased explanatory value of the recommendation algorithm.

## Interactive course recommendation

**Fig. 1:**
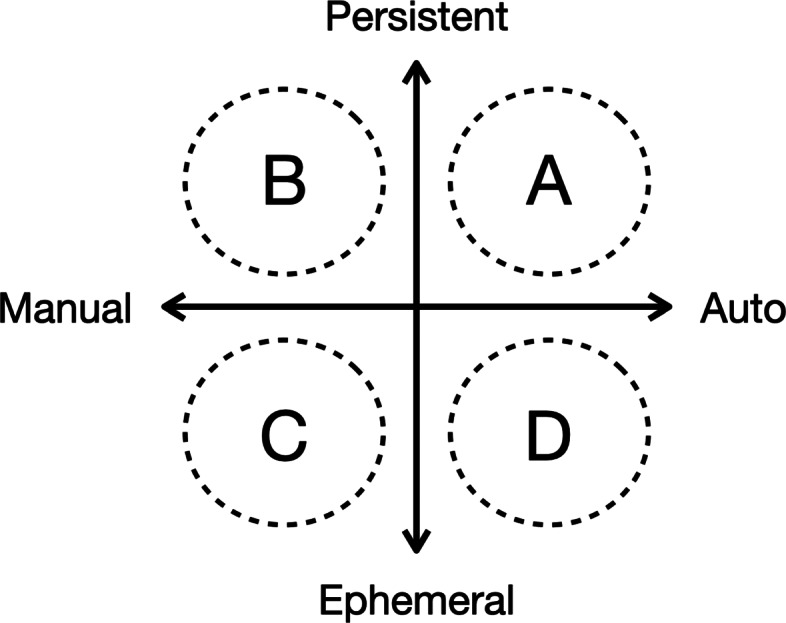
Recommendation taxonomy

Historically, researchers have focused on making recommendations more accurate, with the implicit assumption that more accurate equals more useful (McNee et al. 2006). However, it is difficult to know what kinds of recommendations are good and useful to users without the broader context of the users, which could be revealed through interaction. A user-focused taxonomy is helpful for us to think about the design of recommendation systems from a user-centric perspective as shown in Fig. 1 (Schafer et al. 1999; Lee and Brusilovsky 2007).

The two axes of the taxonomy are the degree of automation and the degree of persistence in the recommendation. Most course recommendation systems that rely on students’ historical course enrollment data can be seen as automatic and persistent recommendations (quadrant A), that is, every time users use the system, data are collected automatically to suggest information fitting into users’ interest. For manual and persistent recommendations (quadrant B), users must spend some manual efforts, i.e., type in several items of interest or answer some questions to make a recommendation, and their behaviors are recorded for the next recommendation. Manual and ephemeral recommendations (quadrant C) is similar to information seeking mechanisms such as search and filter which use users’ input to provide suggestions to match their current interests and requirements, but user behaviors are not recorded for the next recommendation. However, it also needs users to mentally form a more or less specific search term which may be difficult in large or unknown domains. Automatic and ephemeral recommendation (quadrant D) is not dependent on users. Hence, results are the same for all users, such as popular recommendations.

Based on the user-focused taxonomy, we incorporate a suite of technological components to design our interactive course recommendation system, thereby allowing users to explore information in multiple ways. Another design goal is to support the exploration and explanation of the recommendation processes for better engagement, experience, and acceptance.

### System design

**Fig. 2:**
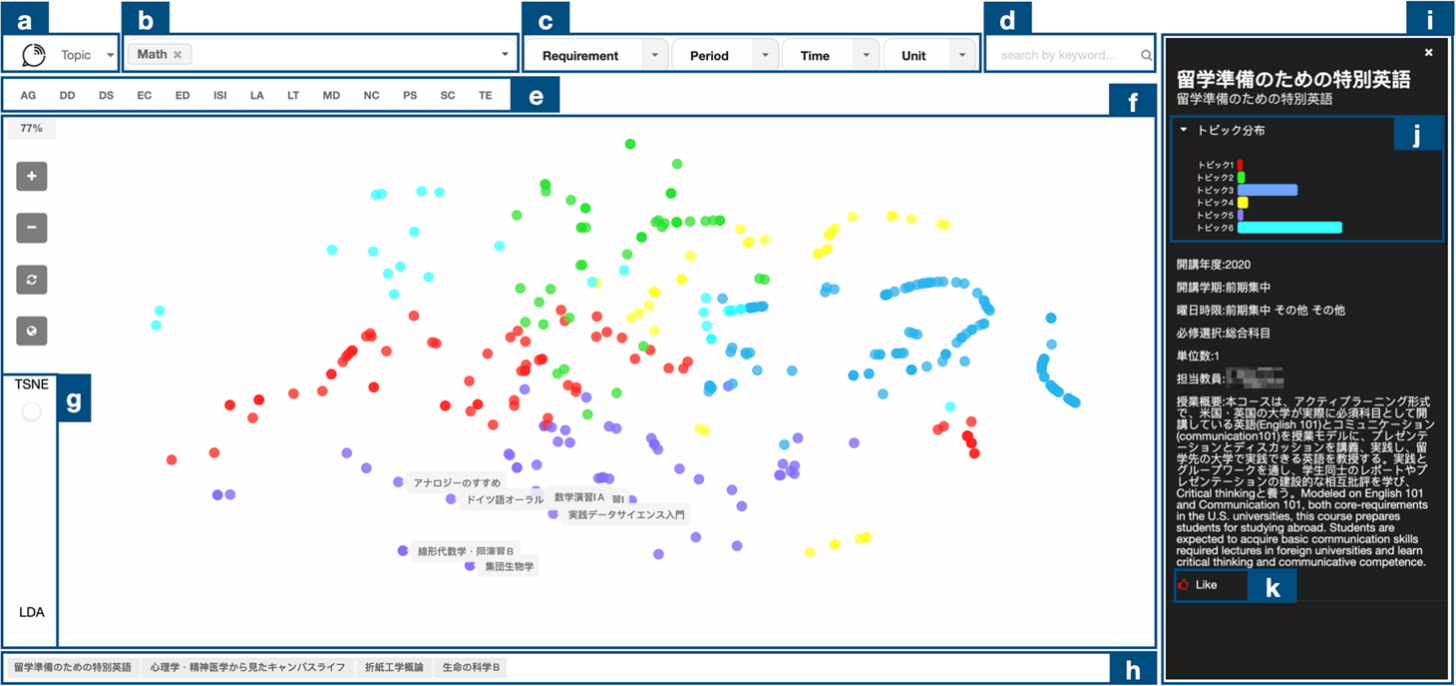
The screenshot of the CourseQ interface. The interface supports the exploration of recommended courses in left and details in right. Left: (a) Navigation tab, (b) Keyword input area, (c) Filters, (d) Search bar, (e) Department information, (f) Scatter plot, (g) Visual shift slider, (h) “Like” list. Right: Detail of the course. (i) Information sidebar, (j) Topic histogram, (k) “Like” button. (Some text is in Japanese, and the instructor name has been pixelated for privacy protection)

In this section, we present CourseQ, a web-based interactive course recommendation system to help students with different information needs to find suitable courses. Figure 2 shows the screenshot of the CourseQ interface, and we will introduce each component in the next subsections. Students can search and filter the courses based on their interests (quadrant C in Fig. 1), check popular courses (quadrant D in Fig. 1), and obtain personalized recommendations (quadrant B in Fig. 1) using CourseQ. By applying a topic model to visualize courses in the latent space, our system also allows students to explore large-item spaces while providing a high level of user control and transparency. Our interface design follows Shneiderman’s visual information seeking mantra by providing an overview, allowing zooming and panning the visualization, allowing filtering based on requirements, and toggling the visibility of details (Shneiderman 1996).

#### Navigation and keywords input

Figure 2 (a) is the Navigation tab; a student could click it to see all topics and related keywords predetermined by our topic model respectively. Figure 2 (b) is the input area. To receive course suggestions, the student could construct their interest by selecting keywords via an interactive drop-down list into the system. Keywords that the student selects represent the student’s academic interests and will be used as a seed for recommendations. After the student selected keywords, the course recommendation system will identify the course whose content best matches the topics corresponding to the selected keywords.

#### Dynamic scatter plot

We used a dynamic scatter plot as it is an intuitive way to show multidimensional data. The interactivity afforded by the dynamic scatter plot visualization could help users make sense of the recommendation results and explore alternatives within the latent space. The interface presents each item (a course) as a circle node on the canvas in a 2D layout (Fig. 2 (f)). We color each course node according to the algorithmically generated topics for a visual explanation. Our interface support zooming and panning the visualization, and the algorithmic layout of the nodes can be shifted across two models (e.g., T-SNE and LDA) by using the slider (Fig. 2 (g)).

Based on the student’s interest associated with their selected keywords, the system recommends courses for the student and shows them in the latent topic space. Recommendations are displayed as the corresponding course nodes and their labels are highlighted within the visualization.

#### Search and filter component

On the upper right side, Fig. 2 (d), students can use a search box with auto-completion to find courses. This is suitable for situations when the student at least has a vague idea of the desired courses and a clear search goal has been formed. The system also combines the benefits of filters to make recommendations to satisfy constraints or requirements from different students. The requirements of the degree program and other components such as period, time, and unit contain check-boxes to filter out and control the recommendations while users interacting with the visualization (Fig. 2 (c)). There are complex constraints and contexts that have to be considered by students for graduation. For example, a student is looking for a course that opens in the following semester with two units to satisfy the requirement of graduation, such a recommendation system that offers filters can be helpful for the student to find appropriate results. In another example, a student dislikes waking up in the early morning so he/she would like to filter morning classes out. The visualization represents all available courses with a circle node and courses that do not satisfy the requirement will be hidden. Our system provides such features as we believe that the system is more useful if it recommends courses not only based on interests but also based on other requirements of students.

#### Department feature

We also have the department information extracted from historic enrollment data in the system (Fig. 2 (e)). Upon clicking on one of the buttons that represent different departments, popular courses within this department will be shown. Students are able to explore popular courses for convenience’s sake and it can be helpful to figure out the similarity or differences among departments, comprehend the course selection pattern, and build their learning path.

#### Information provision and explanation

Upon clicking on the circle node of the recommended course, various information about the course is shown in the right-sidebar (Fig. 2 (i)), students can explore official information provided by the university such as course period, instructor, time, and course descriptions, etc. The explanation is important to increase perceived recommendation quality and trustworthiness (Bigras et al. 2018; Kunkel et al. 2019). To explain why a course is recommended, we used a grouped bar chart, as seen in Fig. 2 (j), which shows the topic distribution of the selected course. The colors of the bars match those of the circle nodes from the visualization in order to show their relations. With the bar charts, students can compare the topic distributions among different courses to help their decision-making processes.

#### “Like” list

The student can click on the button to “like” a course or cancel it as seen in Fig. 2 (k). On the bottom of the interface (Fig. 2 (h)), students can see the list of the courses they liked. In this part of the interface, they can also click on the course to check the detailed information or edit their list to generate personalize results. This component allows a user to provide feedback on recommendations, and iterate until the user validates one or more recommendations as being useful.

Overall, to make the system flexible and useful in different contexts of use (e.g., for the presence of different students, or cold start situations), the recommendation process is entirely based on explicit user input given during a session. On one hand, students can search and filter the courses based on their requirements and check popular courses. On the other hand, they could select keywords and “like” courses to obtain personalized recommendations in an iterative way. In addition, our visualization and topic distribution provide exploration and explanation for students, which help them for better decision-making.

### Visual design

In order to display different courses in the latent space, we collected and analyzed data for 380 courses in our university that opened this year. By employing a topic model, we get a multi-dimensional vector representation for each course. Finally, Linear Discriminant Analysis (LDA) and T-Distributed Stochastic Neighbor Embedding (T-SNE) were used to reduce the dimensionality of these vectors to the 2D layout. We will describe the details in the following subsections.

#### Topic modeling

We use the Latent Dirichlet Allocation generative probabilistic model to fit a topic model to the course data collected from the syllabus of our university. Topic models have been widely used in various kinds of text mining tasks. It is a series of algorithms used to discover the “topics” from a set of documents. Topic modeling provides a way to define a topic as a probability distribution over finite set words (in this case, the vocabulary of the course data) in a fixed vocabulary and represent a document (i.e., a course data document) as the distribution over topics. Thus, all topics are present in each course but with different weights.

**Fig. 3:**
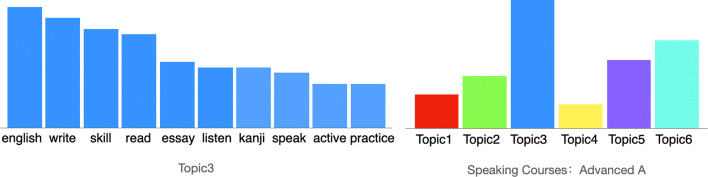
Keyword distribution in topic3 and topic distribution in the course Speaking Courses: Advanced A

In order to understand the relationships of different courses, we extract the text content of collected course data after filtering irrelevant content and employ topic modeling to give a latent representation for each course. By employing topic modeling, we obtain a k-dimensional vector representation for each course where k is the topic number. The topic model helps us to subdivide coarse course data into multiple topics which provides a way to represent the details of course content. Figure 3 shows the word distribution in a topic and the topic distribution in a course based estimated by the topic model fitted on the course data. Topic 3 corresponds to language study and the course Speaking Courses: Advanced A is mainly characterized by topic 3. Since topics act as an intermediary between the keywords entered in the system and the course data, students only need to choose keywords that characterize topics in the course. The use of keywords and topics allows students to focus on their keywords/topic-level interests when searching for courses without forcing them to directly interact with the specificities of each individual course. Moreover, at many universities, conceptually similar courses exist across departments but use widely differing disciplinary vernacular in their catalog descriptions, making them difficult for students to search for and to realize their commonality. We propose that by tuning a vector representation of courses learned from the syllabus data, we can capture enough implicit semantics of the courses to abstractly and accurately construe similarity.

#### Visualization of the course distribution in the latent space

**Fig. 4:**
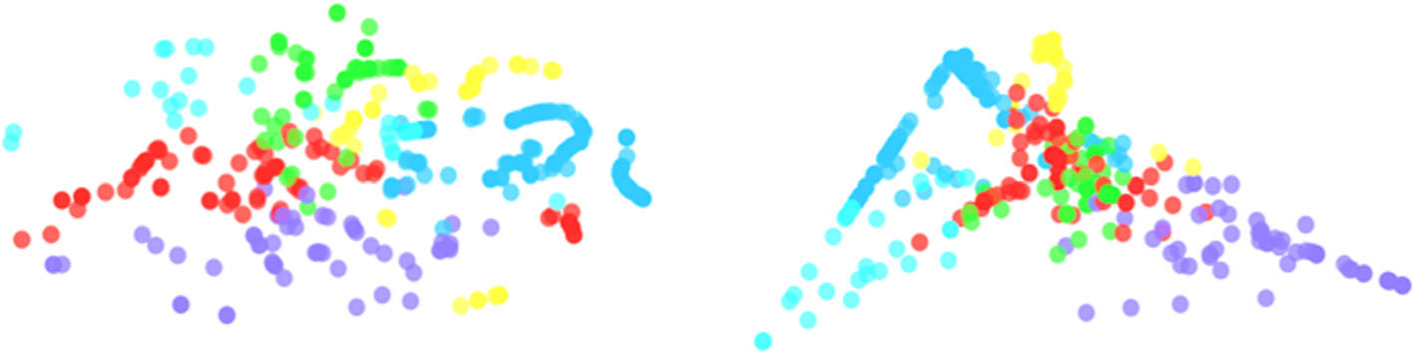
2D layouts: the layout could be continuously shifted from T-SNE distribution (left) to LDA distribution (right) by a slider. The nodes are colored based on topics

The latent representation of course content provides us a convenient way to show the relationships among courses which is an important measurement in our recommendation system. Different methods (LDA and T-SNE) are used to reduce the dimensionality to a 2D layout shown in Fig. 4. We use two different methods and allow students to use the layout based on T-SNE distribution, LDA distribution, or a mixture of both by using a slider.

First, the interface displays the course relevance in two dimensions layout. It helps the student to understand the course content according to its topic distribution. Second, the node is color-coded in different topic features; therefore, the student can perceive the tendencies of the course recommendations based on their coloring. As a result, the visualization interface can be used for course exploration and also as a recommendation explanation.

### Recommendation algorithm

**Fig. 5:**
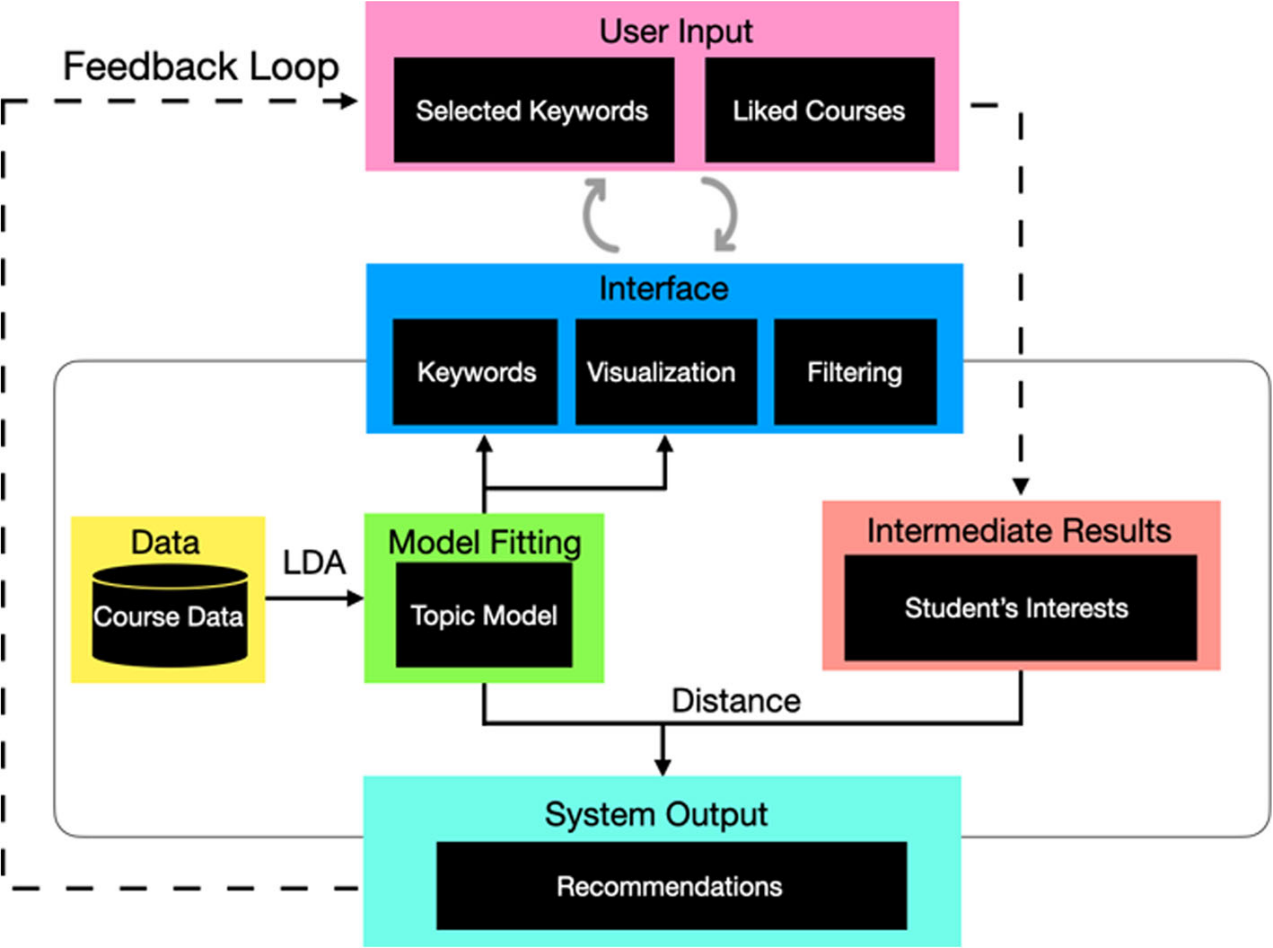
Architecture of the course recommendation system

Figure 5 shows the architecture of our course recommendation system. Our system recommends courses based on the student’s interest corresponding to the topic distribution. The student’s interest is extracted from the keywords that he/she select and courses he/she like while exploring the system. The vector of courses and keywords, based on course content and topic distribution, is calculated offline and stored in two separate data structures. The student’s “like” list is also important information for the system to give more personalized results. Every time the student “liked” a course while browsing the recommendation result or exploring the visualization, it will be added to the “like” list automatically to calculate the student’s interest together with the selected keywords. Also, students could edit their “like” lists conveniently, which allows them to provide immediate feedback and control the system to generate a more personalized result. The recommended courses are ranked based on their similarity to the student’s interest. To this end, we computed the Euclidean distance between the vector of the student’s interest and the vector of each course. We will highlight the top ten as recommended courses and courses around recommendations are all potential candidates for students to explore because they are considered to reflect the latent topics derived from their inputs.

## Evaluation

Evaluating interactive recommendation systems is a non-trivial task because of the complex and potentially diverse interplay between the human participant and the algorithm.

**Fig. 6:**
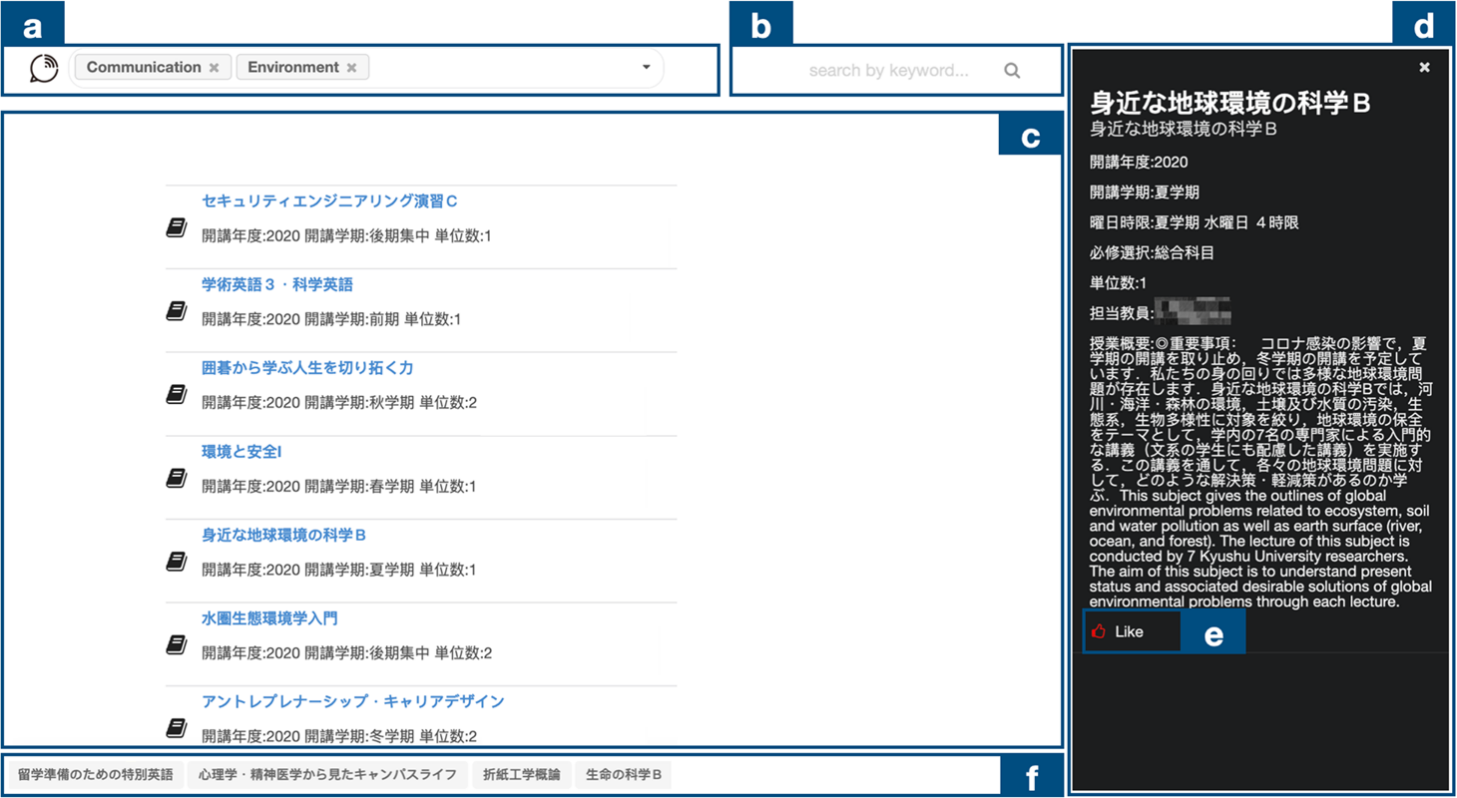
The screenshot of the baseline interface. The interface shows recommended courses in left and details in right. (a) Keyword input area, (b) Search bar, (c) Recommendation list, (d) Information sidebar, (e) “Like” button, (f) “Like” list. (Some text is in Japanese, and the instructor name has been pixelated for privacy protection)

We developed a baseline recommendation system that uses the same algorithm, values, and dataset as CourseQ to perform a comparative evaluation of the effectiveness and quality of the proposed system. Figure 6 illustrates the design of the interface with the baseline system. Considering the fairness of the comparison, we implemented all features of CourseQ. Students can search for courses of interest, get recommendations by selecting keywords, click the recommended course to check details with the information sidebar, and click the button to like and save a course he/she is interested in. However, the proposed visualization and the filter component are removed from the baseline interface. The topic distribution component which acts as an explanation for users is not provided in this interface either. Instead, a conventional ranked list is used as a way of presenting recommendation results.

We hypothesize that the visualization and explanation in CourseQ increasing users’ trust and understanding in the results, and users would give CourseQ better ratings in terms of user-centered aspects than baseline. Moreover, we expect that the proposed functionality in CourseQ could help students with their diverse information needs to find relevant courses more easily in different contexts.

### Participants

After obtaining IRB approval, we recruited 32 participants (22 males and 10 females; 12 undergraduates and 20 graduate students) for the user study. The participants came from different departments of our university; their ages ranged from 19 to 28 (M=25.5, SE=0.39). All of them had prior experience of course selection in a university environment at least once. Due to the Covid-19 situation, the study was conducted fully online in a one-on-one form, the time used for each participant is about 1 h. We used online meeting software (Zoom) to communicate with our participants. Also, we asked them to share the computer screen so we could record all the interaction behaviors during the experiment.

### Experimental setup and data collection

Participants used their computers and a common web browser to access our system. The two different interfaces were tested in a within-subject design. To remedy the influence in the first trial on the second, the first half of the participants will use the CourseQ interface and then use the baseline interface. The other half uses the baseline interface first, and then CourseQ.

After a brief introduction to the experiment and the systems, we asked participants to fill out a questionnaire to collect demographic and personal data, we also asked about their experiences of course selection, their familiarity and knowledge regarding recommendation systems, and if they have a clear goal in relation to their learning. Then, participants were asked to freely interact with the interface to find relevant courses matching their personal interests. They could use all features of the respective interface and there were no time restrictions. They were asked to select at least five courses they were interested in. After performing the tasks, the participants filled out a questionnaire with the 5-point Likert scale (1—completely disagree, 5—completely agree) that measured different aspects of the recommendation systems using the ResQue framework (Pu et al. 2011). We also collected logging data to analyze user interactions with the various elements of the interface during the experiment. Finally, we conducted a qualitative interview with participants to analyze their opinions about the two systems.

## Results

We show our results in the following five subsections. We first present results of the questionnaire focusing on user-centered aspects of the two systems; we also analyzed the results considering demographic and personal characteristics. Next, we present qualitative results based on the interview. In the third subsection, we provide the details of participants’ interactions with the two interfaces to understand how the design affected the interaction behaviors of different users. Next, we present the results in terms of the diversity and coverage of the system. Finally, to better understand the mediation effects across the two interfaces, we present the result of structural equation model (SEM) analysis (di Sciascio et al. 2018) to inspect the impact of the proposed interface on the user experiences.

### Analysis of post study questionnaires

**Fig. 7:**
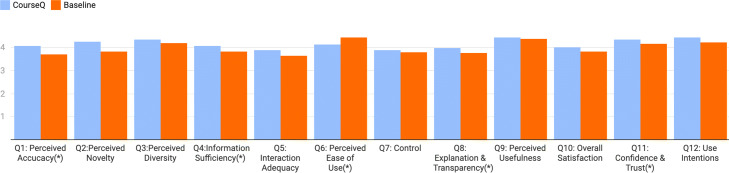
Analysis of the questionnaire results. (Significance level: (*) p < 0.05)

To compare user feedback, we analyze the responses of the questionnaires using paired sample t-tests. Figure 7 presents the subjective feedback from the participants to compare the two interfaces. Overall, participants perceived the CourseQ interface effective. The CourseQ interface received a significantly higher rating for the following four aspects: Perceived Accuracy (Q1, M _CourseQ=4.06, M _Baseline=3.69), Information Sufficiency (Q4, M _CourseQ=4.06, M _Baseline=3.81), Explanation & Transparency (Q8, M _CourseQ=3.97, M _Baseline=3.75), Confidence & Trust (Q11, M _CourseQ=4.34, M _Baseline=4.15). We are using the same recommendation algorithm in both systems, but the perceived accuracy increases when the user interface allows for visualization and explanation. With regard to the Perceived Ease of Use, the baseline interface scored higher than the CourseQ interface (Q6, M _CourseQ=4.13, M _Baseline=4.44). It might be because of a relatively high-effort needed for participants to use the unfamiliar CourseQ interface. For other questions, although without statistical significance, the CourseQ interface scored higher than the baseline interface: Perceived Novelty (Q2, M _CourseQ=4.25, M _Baseline=4.13), Perceived Diversity (Q3, M _CourseQ=4.34, M _Baseline=4.19), Interaction Adequacy (Q5, M _CourseQ=3.88, M _Baseline=3.63), Control (Q7, M _CourseQ=3.88, M _Baseline=3.78), Perceived Usefulness (Q9, M _CourseQ=4.44, M _Baseline=4.37), Overall Satisfaction (Q10, M _CourseQ=4, M _Baseline=3.81), and Use Intentions (Q12, M _CourseQ=4.44, M _Baseline=4.21).

**Fig. 8:**
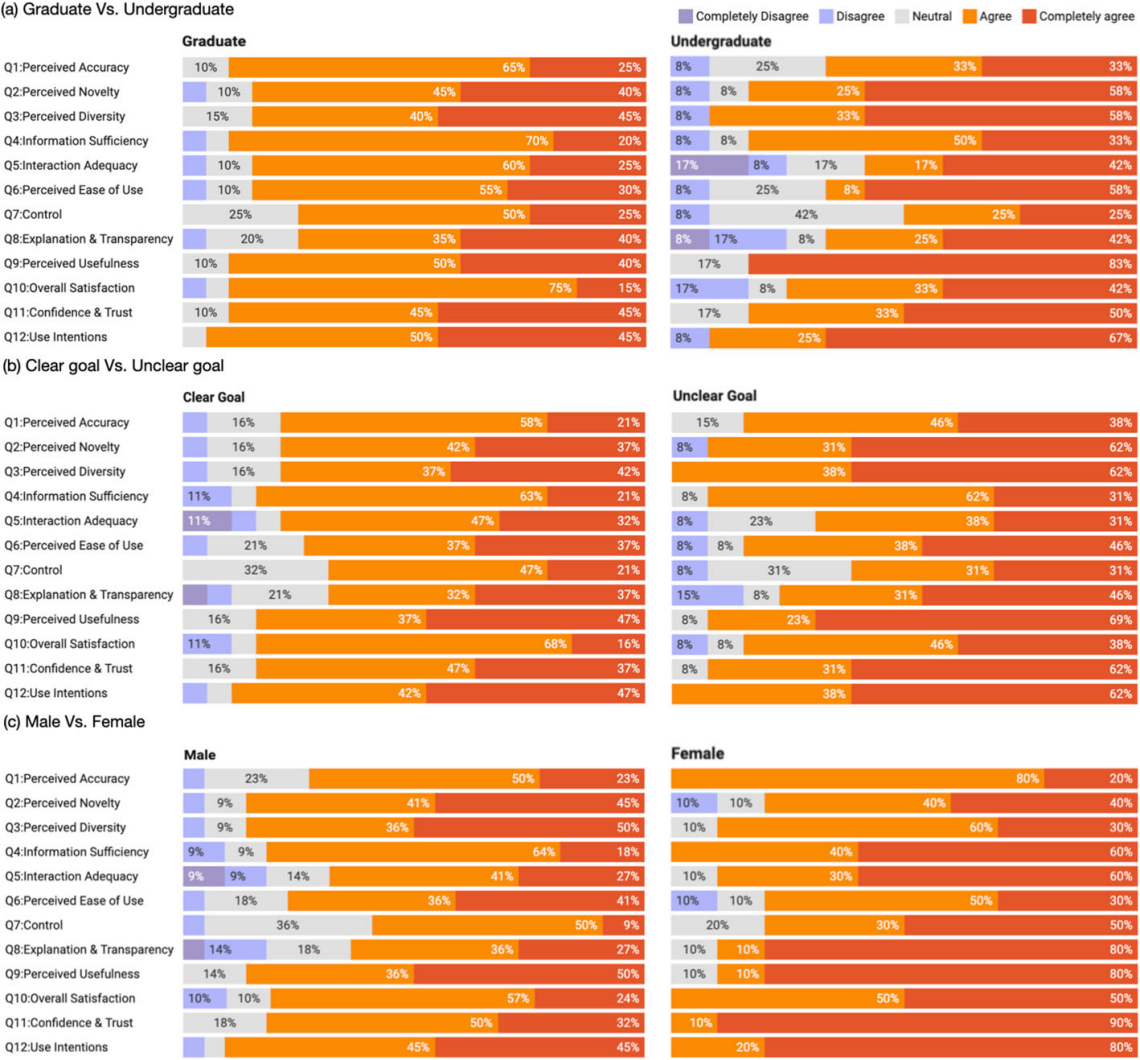
Questionnaire results: **a** Graduate vs. undergraduate. **b** Clear goal vs. unclear goal. **c** Male vs. female

We also analyzed the results with respect to demographic and personal characteristics using independent samples t-test. Overall, CourseQ received positive feedback for all questions across the different backgrounds and characteristics groups (Fig. 8). Undergraduate students (N=12) are significantly positive regarding Perceived Usefulness (Q9) than graduate students as shown in Fig. 8a. The qualitative interview suggests that undergraduate students consider CouresQ a useful tool in exploring their academic interests and finding suitable courses. Graduate students (N =20) were a bit less positive for these questions compared to undergraduate students. They provided detailed suggestions for improving the interface, such as providing information about grade distribution and course ratings by previous students. However, they are significantly positive regarding Interaction Adequacy(Q5). Figure 8b shows the results comparing the students with clear learning goals (N=19) and the students without clear learning goals (N =13). Students without clear learning goals gave significantly positive answers for Perceived Usefulness (Q9), Overall Satisfaction (Q10), Confidence & Trust (Q11), and Use Intentions (Q12). These questions are relevant to the visualization capability of CourseQ, which is designed to empower students to explore and understand the recommendations and find the right courses for them. Although not significant, Perceived Accuracy (Q1) and Perceived Diversity (Q3) also were higher for the students without clear learning goals. In contrast, the results for Perceived Accuracy (Q1) and Confidence & Trust (Q11) were a bit lower for students with clear goals. The results in Fig. 8c show gender differences regarding different aspects. Generally, female students (N=10) gave significantly higher scores for Interaction Adequacy (Q5), Control (Q7), Explanation & Transparency (Q8), and Confidence & Trust (Q11) than male students (N=22).

### Qualitative analysis

Participants were asked to provide feedback on their experience and give suggestions for improving two systems in the qualitative interviews. The positive and negative interview feedback could be summarized into several categories; we will discuss them separately. The interviews were carried out in Chinese and Japanese; we translated the transcriptions into English.

#### Explanation

Many participants enjoyed using the CourseQ interface to discover courses. Eleven participants explicitly mention the explanations as a key enabler for finding desired courses. Participant 30 said that “It is a novel tool to help find courses, I could understand the relationships between courses easily”. Participant 2 indicates that “The explanations help me to understand topics of a course”.

#### Visualization

The visualization seems to be another important aspect, as it was mentioned by nine participants. Participant 4 indicates that “It is a good way to visualize the course data and offer the overview of the latent space for users”. Participant 8 indicates that “The visualization is a map to guide me to find good courses and understand why I should take them”. Participant 11 indicates that “It helped introduce me to courses in different departments that I didn’t know before and were very good”. Participant 13 mentions that “The information provided is very detailed, I can easily find courses related to my interest even from other departments”.

#### Department information

Department information represents popular courses in different departments. This is another aspect that is often mentioned by the interviewees, as it helps to explore their academic interests and find suitable courses. Participant 1 indicates that “We can see popular courses in our department, it is really helpful”. Participant 20 mentions that “Department information is really nice, I would like to see more information like showing popular courses for each semester”.

#### Cost to learn

For the negative comments, the relatively high-effort needed to familiarize themselves with the system was mentioned by six participants. Participant 21 indicates that “We can explore rich information provided by the CouresQ system, but it costs time to learn how to use”. Participant 9 indicates that “It is time-consuming to digest all the information provided by the system, but if I already had a very clear information need, I prefer to come to the simple system that gives me suggestions directly”.

#### Information inadequacy

Information inadequacy was mentioned for both interfaces. Many participants indicated that the information provided by the baseline interface is scarce. Participant 31 indicates that “The information provided by the baseline interface is not enough.” Participant 17 mentions “There is a big blank space, use it to provide more details”.

Interestingly, several participants also mention that the CourseQ interface should provide additional information. Participant 14 comments that “Provide more information, such as grade distribution, previous students’ ratings and comments, textbooks, and other references”.

### Interaction patterns

To better understand the use of the system, we logged user interactions with the systems, most of which were clicks on different interface components. We also recorded the amount of time they spent on two interfaces. Table 1 shows the detailed statistics for two interfaces using paired sample t-tests. Overall, the click frequency presented a significant difference between the two interfaces. The participants tended to interact more with the scatter plot visualization in the CourseQ interface (M=65.34) than the ranked list in the baseline interface (M=12.13). This finding is not surprising because the baseline interface lacks the visualization information that pushes the participant to click more to explore within the item space. Moreover, the participants tended to interact more with the information and explanation sidebar in the CourseQ interface (M=23.2) than the baseline interface (M=7.8). Also, there is a significant difference in the time spent on the task between the CourseQ interface (M=542.28) and the baseline interface (M=290.31). This hints that the CouresQ interface could serve as an interactive exploration interface that engages students in the task of finding interesting courses.

**Table 1:** User interaction statistics (Significance level: (*) p<0.05.)

	CourseQ	Baseline	
Component—Behavior	M(SE)	M(SE)	*P*-value
Ranked list—total clicks	-	12.13(6.76)	
Scatter plot—total clicks	65.34(13.55)	-	
Navigation and keywords input—total clicks	11.81(2.29)	12.29(3.42)	
Search and filter—total clicks	25.22(5.26)	2.9(3.72)	*
Department feature—total clicks	7.81(1.71)	-	
Information and explanation—total clicks	23.22(5.59)	7.8(4.2)	*
“Like” list—total clicks	1.63(0.85)	3.2(1.1)	
Time spent—seconds	542.28(105.38)	290.31(56.89)	*

**Fig. 9:**
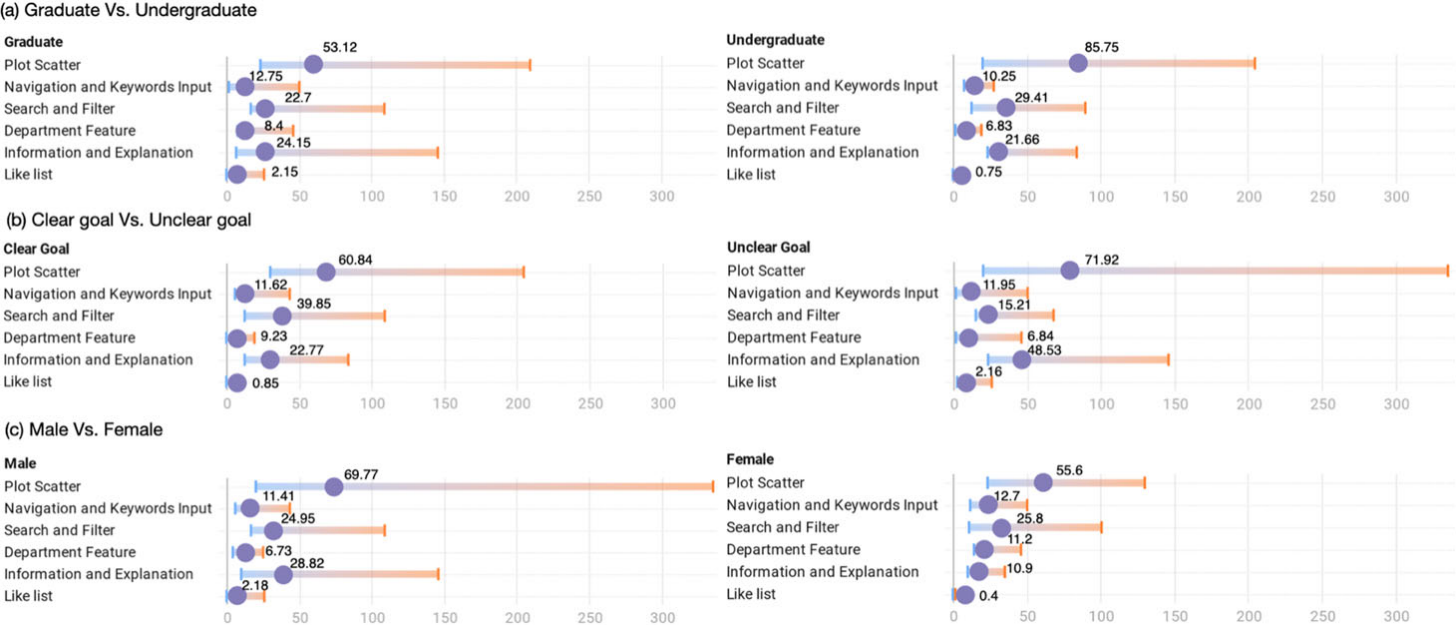
Interaction results: **a** Graduate vs. undergraduate. **b** Clear goal vs. unclear goal. **c** Male vs. female

We also summarize the overview of the interaction traces of participants with respective demographic and personal characteristics in Fig. 9. In general, undergraduate participants interacted more frequently with the scatter plot visualization (M = 85.75) than graduate participants (M = 53.12); see Fig. 9a. Figure 9b shows the interaction patterns comparing the students with clear learning goals and the students without clear learning goals. We can see that students with clear learning goals engaged more with the search and filter component (M = 39.85), while students without clear learning goals interacted more with the scatter plot visualization (M = 71.92). Moreover, when using the interface, these students frequently checked the information and explanation component (M=48.53). This is conceivable since the student with clear learning goals might already have a vague idea of the desired courses or a clear search goal, and the student without clear learning goals might need to start by exploring the relationship among courses to dig more information by using CourseQ. There is no significant difference between female students and male students. We can observe that male students engaged more with the scatter plot. Female students tended to check more about the department feature component. They reported that department information represents popular courses in different departments, and is useful for them. They also suggested adding more information like showing popular courses for each semester.

### Diversity and coverage

Promoting diversity in recommendation systems is one of the most important topics in the area, particularly helpful in preventing the creation of filter bubbles (Steck et al. 2015). To understand the effect of interaction functionality in a visual interface on the diversity among recommendations, we compared the number of unique courses checked per user in each interface. Moreover, we measured the percentage of courses that have been checked once by participants to study the coverage of systems. As coverage describes the ability of exploration, we assume that the visualization and explanation of CourseQ could improve user’s engagement and help to explore the item space.

The result in Table 2 shows that participants who use the CourseQ interface checked more unique courses than participants who use the baseline interface. Also, as we expected, the CourseQ interface outperforms in terms of coverage. The results highlighted the exploration ability of the CourseQ interface. That is, the use of interactive visualization pushes the participants to click more to explore within the item space.

**Table 2:** Number of unique courses per user and overall coverage

	No. of unique courses per user	Coverage
	M(SE)	Percentage
CourseQ	23.97(4.23)	53.18%
Baseline	9.38(1.13)	37.58%

### Structural modeling

Since our system combines a recommendation algorithm, an interactive interface, and subjective experiences of participants in the experiment, there are many variables that interact with each other. To study these interactions and better understand the causal and mediation effects across the two interfaces, we applied the structural equation model (SEM) to inspect the effects of the proposed interface. There are five elements in the model:

- Objective System Aspect (OSA): manipulations that were controlled in two interfaces.
- Subjective System Aspects (SSA): factors based on post study questionnaires.
- Personal Characteristics (PC): personal characteristics from pre-study questionnaires.
- Interactions (INT): observed dependent variables from the analysis of the system log data.
- Experience (EXP): the user experience with the system based on post study questionnaires.

**Fig. 10:**
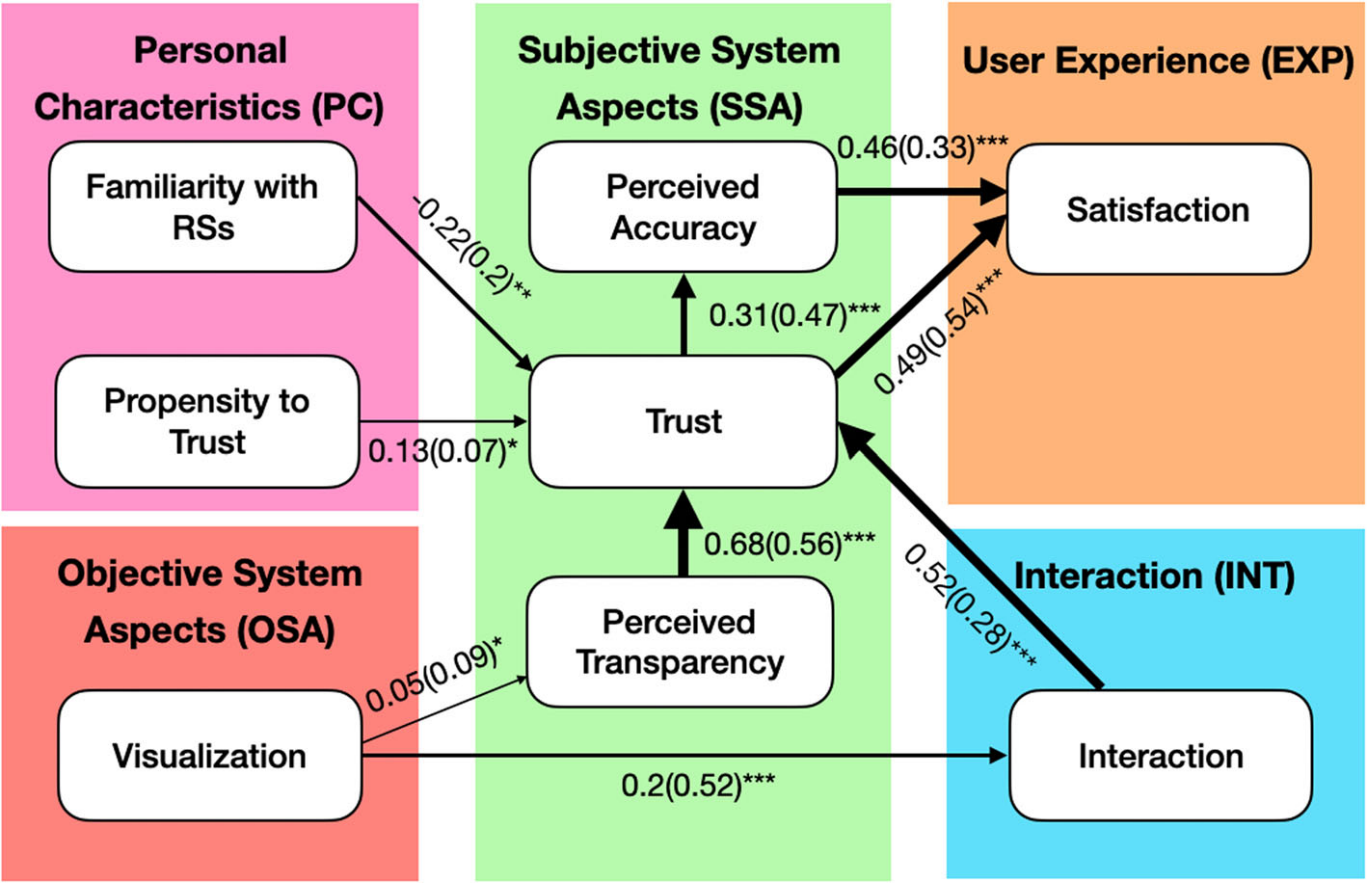
The structural equation model of the experiment. Pathways represent significant relationships, with numbers and thickness indicating regression coefficients (with robust SE). All factors in the model have been scaled to have a standard deviation of 1. Significance levels are *** p<0.01, ** p<0.05, * p<0.1

Figure 10 shows the result of structural model. The model obtained good fit to the data, *χ*^2^ (19) = 22.99, p = 0.23, CFI = 0.969, TLI = 0.954, RMSEA = 0.058 with 90% CI = [0, 0.129]. Regression pathways report standardized coefficients and robust standard errors. In this representation, edge thickness highlights the stronger effect sizes and effect direction indicates positive or negative.

#### Personal characteristics

Personal characteristics of a user are known to influence their experience with a system; previous studies on recommendation systems have shown a relationship between the inherent user’s propensity to trust and its final perception of the system (Parra and Brusilovsky 2015; Knijnenburg et al. 2012; Knijnenburg et al. 2011). The relations in our structural model also indicates that propensity trust plays an important role in how users perceive recommendations. However, users acknowledging high familiarity with recommendation systems have a negative effect on perceived trust.

#### Trust

Except for the user’s propensity to trust, the model also reveals a large effect of other factors on trust. More specifically, trust seems to be greatly influenced by the perceived transparency and their interaction level with the system. First, the CourseQ interface with interactive visualization and explanations has a direct positive effect on users’ perception of transparency, and the perception of transparency influenced trust in the system. Also, perceived trust is greatly influenced by the interaction level of users directly. When participants interacted with the CourseQ interface, they performed significantly more interactions compared to the baseline interface. In turn, more interactions have a positive effect on the user’s trust. Interestingly, even if the visualization causes an improvement in perceived transparency, it has no direct significant effect to help the user to gain trust in the interface compared to the interaction. That is, users need time to interact with the system and learn how to use it to fully understand the system and trust it.

#### User satisfaction

We can observe that the effect of the interface on user satisfaction is not direct but transferable through trust and perceived accuracy. As we highlighted the importance of interactions for the system to understand user needs and thus provide more accurate course recommendations. The CourseQ interface with visualization empowers users to interact more with the system, and the interaction influenced trust in the system. Moreover, trust has a positive effect on perceived accuracy. Then, perceived accuracy and trust both lead to higher user satisfaction.

From the statistical evidence, we can corroborate that higher satisfaction is a result of not only a good-performing system but also other aspects in the interface such as trust and interaction. To sum up, although two systems use the same algorithm, values, and dataset, only the CourseQ interface provides visualizations and explanations. The evidence suggests that the CourseQ interface outperforms the baseline interface. In conclusion, the CourseQ system with visualizations and explanations influenced the perception of transparency and engage users to interact more with it and make users feel satisfied with the system.

## Discussion

In this section, we link the results of the questionnaire to the interaction patterns of different users; we also discuss the implication for the design of future interactive course recommendation systems.

### Connecting user behavior and feedback

Responses to the questions indicate that students value the use of interactive visualization techniques to find suitable courses. The overall results show that CourseQ is potentially useful to the students, thereby helping them make better-informed course selections. Also, most participants indicated that they feel confident about and trust CourseQ, and will use it again. Visualizations and explanations seem to contribute well to better support of user empowerment. Feedback from different participants shows that they all have a better understanding of the course recommendations by being able to see the topic distribution and course relationship at the same time. The user interaction indicates that all participants engaged more with CourseQ, with a mean number of clicks above 135 for all users, although some users did not use all features to interact with it. The results also indicate that participants interacted more frequently with the scatter plot visualization; it enables them to explore more diverse recommendations and understand the relevance of different courses. In addition, we can observe interesting differences between different participants. Students without learning goals gave relatively positive answers for Perceived Accuracy and Perceived Diversity. We can observe that they interacted more with the scatter plot visualization, information, and explanation component to dig more information by using CourseQ. For students with clear goals, they reported the relatively high-effort needed to learn to use CourseQ. When using CourseQ, they engaged more with the search and filter component, try to get the right recommendation in a more direct way. As a further result, design based on the user-focused taxonomy, CourseQ can meet the diverse needs of different students by incorporating various technologies to allow users to explore information in multiple ways, from popular recommendation to highly user-adaptive recommendation, but the interaction may need further simplification for some users.

### Design implications

We presented CourseQ, an interactive course recommendation system to help the exploration and explanation of the recommendation processes, our experimental results could be helpful for the design of future course recommendation systems. First, the user should be able to control recommendations by providing feedback and filter out the information by prioritizing specific items of interest. The interaction is one of the most important features of our system; it ranges from zooming and panning the visualization to explore the courses. Besides, users can control the recommendations by clicking on the “like” button to provide feedback and iterate until the user validates one or more recommendations as being useful. Second, recommendations should be explained, and details should be provided on-demand. In CourseQ, recommended courses will be highlighted in the visualization, which helps users understand how those courses are related to each other. The explanation and exploration are further supported by providing official course information and show the topic distribution bar chart for the recommended item. In addition, a useful recommendation system should generate recommendations for users with different information needs. We incorporate various technologies to design our interactive course recommendation system based on the user-focused taxonomy. CourseQ can meet the diverse needs of different students by incorporating various technologies to allow users to explore information in multiple ways, from popular recommendation to highly user-adaptive recommendation.

## Conclusion

In this paper, we presented CourseQ, a course recommendation system that integrates interactive visualization and recommendation techniques to help the exploration and explanation of the recommendation processes through an interactive interface. Besides the integration of recommendation techniques with interactive visualization, a major goal of this work has been to meet diverse needs from different students to allow them to explore information in multiple ways based on their situational requirements. We designed the system based on the user-focused taxonomy to achieve this goal.

A within-subject user study (N=32) was presented to evaluate the interaction and recommendation concept of CourseQ, compared with a baseline recommendation system. Our key results have shown that interface design and a certain combination of interactive features improve perceived recommendation accuracy, as well as user satisfaction with the recommendation system. Moreover, our proposed interface can meet the diverse needs of different users by incorporating various technologies to allow users to explore information in multiple ways. We noticed that some participants may find the baseline system is easier to use than CourseQ. This finding helps us to realize the user preference for adopting the interface with lower learning efforts. Overall, the CourseQ was perceived as effective in terms of recommendation quality as well as usability, but the interaction may need further simplification for some users. In addition, our study suggested opportunities to extend CourseQ in different ways by incorporating more information, such as grade distribution and course rating, and visually representing complex constraints and requirements of course types and credits.

There are some limitations to this work that needs to be articulated. First, partly due to the Covid-19 situation this year, the scale of reported user studies is relatively small, and it may decrease the statistical power of the finding. Second, our qualitative evaluation focused on the user’s perception of the interaction concept and the quality of the results, which could be complemented with additional quantitative objective measurements.

## Data Availability

Data and material are not available as our consent forms did not include information regarding sharing data outside of the research study.

## References

[CR1] Aher S.B., Lobo L. (2013). Combination of machine learning algorithms for recommendation of courses in e-learning system based on historical data. Knowledge-Based Systems.

[CR2] Alkan, O., Daly, E.M., Botea, A., Valente, A.N., Pedemonte, P. (2019). Where can my career take me? harnessing dialogue for interactive career goal recommendations. In *Proceedings of the 24th International Conference on Intelligent User Interfaces*, (pp. 603–613).

[CR3] Andjelkovic, I., Parra, D., O’Donovan, J. (2016). Moodplay: Interactive mood-based music discovery and recommendation. In *Proceedings of the 2016 Conference on User Modeling Adaptation and Personalization*, (pp. 275–279).

[CR4] Parra D., O’Donovan J., Andjelkovic, I. (2019). Moodplay: Interactive music recommendation based on artists’ mood similarity. International Journal of Human-Computer Studies.

[CR5] Bendakir, N., & Aïmeur, E. (2006). Using association rules for course recommendation. In *Proceedings of the AAAI Workshop on Educational Data Mining*, (Vol. 3, pp. 1–10).

[CR6] Bigras, E., Jutras, M.-A., Sénécal, S., Léger, P.-M., Fredette, M., Black, C., Robitaille, N., Grande, K., Hudon, C. (2018). Working with a recommendation agent: How recommendation presentation influences users’ perceptions and behaviors. In *Extended Abstracts of the 2018 CHI Conference on Human Factors in Computing Systems*, (pp. 1–6).

[CR7] Bostandjiev, S., O’Donovan, J., Höllerer, T. (2012). Tasteweights: a visual interactive hybrid recommender system. In *Proceedings of the Sixth ACM Conference on Recommender Systems*, (pp. 35–42).

[CR8] Bostandjiev, S., O’Donovan, J., Höllerer, T. (2013). LinkedVis: Exploring social and semantic career recommendations. In *Proceedings of the 2013 International Conference on Intelligent User Interfaces*, (pp. 107–116).

[CR9] Burke, R. (2010). Evaluating the dynamic properties of recommendation algorithms. In *Proceedings of the Fourth ACM Conference on Recommender Systems*, (pp. 225–228).

[CR10] Chatzimparmpas A., Martins R.M., Jusufi I., Kerren A. (2020). A survey of surveys on the use of visualization for interpreting machine learning models. Information Visualization.

[CR11] Chi, E.H. (2004). Transient user profiling. In *Proc. Workshop on User Profiling*, (pp. 521–523).

[CR12] di Sciascio, C., Brusilovsky, P., Veas, E. (2018). A study on user-controllable social exploratory search. In *23rd International Conference on Intelligent User Interfaces*, (pp. 353–364).

[CR13] Du F., Plaisant C., Spring N., Crowley K., Shneiderman B. (2019). Eventaction: A visual analytics approach to explainable recommendation for event sequences. ACM Transactions on Interactive Intelligent Systems (TiiS).

[CR14] Du, F., Plaisant, C., Spring, N., Shneiderman, B. (2017). Finding similar people to guide life choices: Challenge, design, and evaluation. In *Proceedings of the 2017 CHI Conference on Human Factors in Computing Systems*, (pp. 5498–5544).

[CR15] Elbadrawy, A., & Karypis, G. (2016). Domain-aware grade prediction and top-n course recommendation. In *Proceedings of the 10th ACM Conference on Recommender Systems*, (pp. 183–190).

[CR16] Elbadrawy, A., Studham, R.S., Karypis, G. (2015). Collaborative multi-regression models for predicting students’ performance in course activities. In *Proceedings of the Fifth International Conference on Learning Analytics and Knowledge*, (pp. 103–107).

[CR17] Gretarsson B., O’Donovan J., Bostandjiev S., Hall C., Höllerer T. (2010). Smallworlds: visualizing social recommendations. Computer Graphics Forum.

[CR18] Gutiérrez, F., Charleer, S., De Croon, R., Htun, N.N., Goetschalckx, G., Verbert, K. (2019). Explaining and exploring job recommendations: A user-driven approach for interacting with knowledge-based job recommender systems. In *Proceedings of the 13th ACM Conference on Recommender Systems*, (pp. 60–68).

[CR19] He C., Parra D., Verbert K. (2016). Interactive recommender systems: A survey of the state of the art and future research challenges and opportunities. Expert Systems with Applications.

[CR20] Herlocker, J.L., Konstan, J.A., Riedl, J. (2000). Explaining collaborative filtering recommendations. In *Proceedings of the 2000 ACM Conference on Computer Supported Cooperative Work*, (pp. 241–250).

[CR21] Hu Q., Rangwala H. (2018). Course-specific Markovian models for grade prediction. Pacific-Asia Conference on Knowledge Discovery and Data Mining.

[CR22] Huang L., Wang C.-D., Chao H.-Y., Lai J.-H., Philip S.Y. (2019). A score prediction approach for optional course recommendation via cross-user-domain collaborative filtering. IEEE Access.

[CR23] Jiang, W., Pardos, Z.A., Wei, Q. (2019). Goal-based course recommendation. In *Proceedings of the 9th International Conference on Learning Analytics & Knowledge*, (pp. 36–45).

[CR24] Jin Y., Cardoso B., Verbert K. (2017). How do different levels of user control affect cognitive load and acceptance of recommendations?. CEUR Workshop Proceedings.

[CR25] Jing, X., & Tang, J. (2017). Guess you like: course recommendation in MOOCs. In *Proceedings of the International Conference on Web Intelligence*, (pp. 783–789).

[CR26] Knijnenburg, B.P., Bostandjiev, S., O’Donovan, J., Kobsa, A. (2012). Inspectability and control in social recommenders. In *Proceedings of the Sixth ACM Conference on Recommender Systems*, (pp. 43–50).

[CR27] Knijnenburg, B.P., Reijmer, N.J., Willemsen, M.C. (2011). Each to his own: how different users call for different interaction methods in recommender systems. In *Proceedings of the Fifth ACM Conference on Recommender Systems*, (pp. 141–148).

[CR28] Konstan J.A., Riedl J. (2012). Recommender systems: from algorithms to user experience. User Modeling and User-Adapted Interaction.

[CR29] Kucher, K., Martins, R.M., Kerren, A. (2018). Analysis of VINCI 2009-2017 proceedings. In *Proceedings of the 11th International Symposium on Visual Information Communication and Interaction*, (pp. 97–101).

[CR30] Kunkel, J., Donkers, T., Michael, L., Barbu, C.-M., Ziegler, J. (2019). Let me explain: Impact of personal and impersonal explanations on trust in recommender systems. In *Proceedings of the 2019 CHI Conference on Human Factors in Computing Systems*, (pp. 1–12).

[CR31] Lee D.H., Brusilovsky P. (2007). Fighting information overflow with personalized comprehensive information access: A proactive job recommender. Third International Conference on Autonomic and Autonomous Systems (ICAS’07).

[CR32] Loepp, B., Herrmanny, K., Ziegler, J. (2015). Blended recommending: Integrating interactive information filtering and algorithmic recommender techniques. In *Proceedings of the 33rd Annual ACM Conference on Human Factors in Computing Systems*, (pp. 975–984).

[CR33] Ma, B., Lu, M., Taniguchi, Y., Konomi, S. (2021). Exploration and explanation: An interactive course recommendation system for university environments. In *Proceedings of the ACM IUI 2021 Workshops*. In press.

[CR34] Ma, B., Taniguchi, Y., Konomi, S. (2020). Course recommendation for university environments. In *Proceedings of The 13th International Conference on Educational Data Mining (EDM 2020)*, (pp. 460–466).

[CR35] McNee, S.M., Riedl, J., Konstan, J.A. (2006). Making recommendations better: an analytic model for human-recommender interaction. In *CHI’06 Extended Abstracts on Human Factors in Computing Systems*, (pp. 1103–1108).

[CR36] Morsomme, R., & Alferez, S.V. (2019). Content-based course recommender system for liberal arts education. In *Proceedings of The 12th International Conference on Educational Data Mining (EDM 2019)*, (pp. 748–753).

[CR37] Morsy S., Karypis G. (2019). Will this course increase or decrease your gpa? towards grade-aware course recommendation. Journal of Educational Data Mining.

[CR38] O’Donovan, J., Smyth, B., Gretarsson, B., Bostandjiev, S., Höllerer, T. (2008). PeerChooser: visual interactive recommendation. In *Proceedings of the SIGCHI Conference on Human Factors in Computing Systems*, (pp. 1085–1088).

[CR39] Osborne J., Simon S., Collins S. (2003). Attitudes towards science: A review of the literature and its implications. International Journal of Science Education.

[CR40] Pardos Z.A., Fan Z., Jiang W. (2019). Connectionist recommendation in the wild: On the utility and scrutability of neural networks for personalized course guidance. User Modeling and User-Adapted Interaction.

[CR41] Pardos, Z.A., & Jiang, W. (2020). Designing for serendipity in a university course recommendation system. In *Proceedings of the tenth international conference on learning analytics & knowledge*, (pp. 350–359).

[CR42] Pariser E. (2011). The Filter Bubble: What the Internet Is Hiding from You.

[CR43] Parra D., Brusilovsky P. (2015). User-controllable personalization: A case study with setfusion. International Journal of Human-Computer Studies.

[CR44] Parra, D., Brusilovsky, P., Trattner, C. (2014). See what you want to see: visual user-driven approach for hybrid recommendation. In *Proceedings of the 19th International Conference on Intelligent User Interfaces*, (pp. 235–240).

[CR45] Polyzou, A., Nikolakopoulos, A.N., Karypis, G. (2019). Scholars walk: A Markov chain framework for course recommendation. In *Proceedings of The 12th International Conference on Educational Data Mining (EDM 2019)*, (pp. 396–401).

[CR46] Potts, B.A., Khosravi, H., Reidsema, C., Bakharia, A., Belonogoff, M., Fleming, M. (2018). Reciprocal peer recommendation for learning purposes. In *Proceedings of the 8th International Conference on Learning Analytics and Knowledge*, (pp. 226–235).

[CR47] Pu, P., Chen, L., Hu, R. (2011). A user-centric evaluation framework for recommender systems. In *Proceedings of the Fifth ACM Conference on Recommender Systems*, (pp. 157–164).

[CR48] Chen L., Hu R., Pu, P. (2012). Evaluating recommender systems from the user’s perspective: survey of the state of the art. User Modeling and User-Adapted Interaction.

[CR49] Ricci F., Rokach L., Shapira B. (2011). Introduction to recommender systems handbook. Recommender Systems Handbook.

[CR50] Schafer, J.B., Konstan, J., Riedl, J. (1999). Recommender systems in e-commerce. In *Proceedings of the 1st ACM Conference on Electronic Commerce*, (pp. 158–166).

[CR51] Shneiderman B. (1996). The eyes have it: A task by data type taxonomy for information visualizations. Proceedings 1996 IEEE Symposium on Visual Languages.

[CR52] Sinha, R., & Swearingen, K. (2002). The role of transparency in recommender systems. In *CHI’02 Extended Abstracts on Human Factors in Computing Systems*, (pp. 830–831).

[CR53] Steck, H., van Zwol, R., Johnson, C. (2015). Interactive recommender systems: Tutorial. In *Proceedings of the 9th ACM Conference on Recommender Systems*, (pp. 359–360).

[CR54] Swearingen K., Sinha R. (2001). Beyond algorithms: An HCI perspective on recommender systems. ACM SIGIR 2001 Workshop on Recommender Systems.

[CR55] Sweeney M., Rangwala H., Lester J., Johri A. (2016). Next-term student performance prediction: A recommender systems approach. Journal of Educational Data Mining.

[CR56] Tintarev N., Masthoff J. (2011). Designing and evaluating explanations for recommender systems. Recommender Systems Handbook.

[CR57] Tinto V. (1997). Classrooms as communities: Exploring the educational character of student persistence. The Journal of Higher Education.

[CR58] Tsai, C.-H., & Brusilovsky, P. (2018). Beyond the ranked list: User-driven exploration and diversification of social recommendation. In *23rd International Conference on Intelligent User Interfaces*, (pp. 239–250).

[CR59] Wong, D., Faridani, S., Bitton, E., Hartmann, B., Goldberg, K. (2011). The diversity donut: enabling participant control over the diversity of recommended responses. In *CHI’11 Extended Abstracts on Human Factors in Computing Systems*, (pp. 1471–1476).

[CR60] Xiao B., Benbasat I. (2007). E-commerce product recommendation agents: use, characteristics, and impact. MIS quarterly.

[CR61] Zudilova-Seinstra E., Adriaansen T., Van Liere R. (2009). Overview of interactive visualisation. Trends in Interactive Visualization.

